# 

**Published:** 1812-01

**Authors:** 


### MEDICAL AND PHYSICAL JOURNAL.

CONDUCTED BY

SAMUEL FOTHERGILL, M.D.

AND

WILLIAM ROYSTON, ESQ.

VOL. XXVII.

FROM JANUARY TO JUNE, 1812.

*Et quoniam variant morbi, variabimus artes;*

*Mille mali species, mille salutis erunt.*

*LONDON:*

PRINTED FOR RICHARD PHILLIPS, 47, LUDGATE-HILL;

BY J. ADLARD, 23, BARTHOLOMEW-CLOSE.

W
1
ME 182

[Entered at Stationers' Hall.]

THE
Medical and Physical Journal.

1 OF VOL. XXVII.] JANUARY, 1812. [NO. 155.

### To the READERS of the MEDICAL JOURNAL.

*THE Proprietor of this Work, having received the sanction of the Editors to appropriate this space in the present Number to the Commercial Interests of the Journal, feels it his duty, in the first instance, to express his deep sense of the substantial and extensive patronage with which the Work has been honored since its commencement. Previous to the period at which he planned this publication in 1797, there had existed other periodical works devoted to Medical Literature; but the temporary or inadequate support they had met with, afforded slender prospects on which to calculate on the permanent establishment of so important a series as the* PRESENT MEDICAL JOURNAL. *The execution presented, however, so much living matter, and the several departments exhibited so many strong appeals to the interest and curiosity of the Faculty, that he found himself agreeably disappointed, and this Journal very soon ranked in point of circulation with the most popular works of its time. Its fame and circulation spread through Europe and America, and it was sought with avidity in those parts of Africa and Asia where modern science and the English language had obtained a footing. In Germany it was regularly translated, and enriched with notes by a learned German Editor; and in America it was almost entirely reprinted at more than one city. Such distinctions and unparalleled success naturally drew upon it envy and competition, and hence that tribe of imitators who have started one after another, only to repent of their piratical attempts, during almost every year since the commencement of the Work.*

*Various changes among the Editors have been the unavoidable consequence of the decease of some, and of those fluctuations in stations and pursuits to which medical men, particularly in London, are more liable than any other class of society. The Proprietor, for himself, shall remark, on this subject, that he has always performed his duty liberally, and with an anxious desire to meet the expectations of the Public, and support the reputation of the Journal. In his present arrangements he estimates the approbation of the Public by the greatly increasing sale, and by the opinions unequivocally expressed of its management by some of the first medical men in the metropolis. In a word, the sanction of the Faculty is demonstrated by the value and variety of the communications; these at the same time increasing the number of readers, and, by a fortunate concurrence, an increase of readers necessarily tending at the same time to add to the number of the communications.*

*Independent of the value of these latter, the readers of the Work cannot fail to have been gratified by the enlargement of the Critical Analysis of Medical Books, and by the attention bestowed on the department of Intelligence which tends so particularly to confer on the Journal the characteristic of being* THE GAZETTE OF THE FACULTY.

*The completion of the* TWENTY-SIXTH VOLUME *serves to render this Journal a* VAST LIBRARY *or* DEPÔT *of Medical Science, Opinions, Improvements, and Discoveries; and, on this occasion, the Proprietor feels it proper to state, that, having recently reprinted some scarce numbers, he is now enabled to supply at the regular price any numbers or volumes wanted to perfect sets. He has also half bound some sets with calf and Russia backs, which for a limited period he offers at the reduced price of* FIFTEEN GUINEAS *per set in calf, or* SIXTEEN POUNDS, TEN SHILLINGS, *in Russia. He will also, for the accommodation of Gentlemen who find it inconvenient to get their numbers bound, exchange perfect sets half bound for clean numbers, allowing a proportionate price for numbers returned; and such and all orders may be addressed directly to* SIR RICHARD PHILLIPS, *No. 47, Ludgate Hill, covering remittance, or through any Bookseller in town or country.*

### MONTHLY CATALOGUE OF MEDICAL BOOKS.

MEDICO-Chirurgical Transactions; published by the Medical and Chirurgical Society of London. Vol. II. 8vo.—Longman and Co.

Gillman's (James) Dissertation on the Bite of a Rabid Animal; being the substance of an Essay which received a prize from the Royal College of Surgeons in London, in the year 1811: 8vo.—J. Callow.

Bionomia. Opinions concerning Life and Health, introductory to a Course of Lectures on the Physiology of Sentient Beings. By A. P. Buchan, M.D. of the Royal College of Physicians, London. 8vo.—Cadell and Davies.

Burns' (Allan) Observations on the Surgical Anatomy of the Head and Neck; illustrated by Cases and Engravings: 8vo.—Longman and Co.

Bell's (John) Discourses on the Nature and Cure of Wounds. The Third Edition, revised and corrected. 8vo.—Longman and Co.

Travers' (Benjamin) Inquiry into the Process of Nature in repairing Injuries of the Intestines, illustrating the Treatment of penetrating Wounds and strangulated Hernia. 8vo.—Longman and Co.

Parkes' (Samuel) Chemical Catechism; with notes, illustrations, and experiments, 8vo.—Lackington and Co.

Moore's (James) Two Letters to Dr. Jones on the Composition of the Eau Medicinale d'Husson. Second Edition, corrected. 8vo.—Johnson and Co.

Syer's (John) Treatise on the Management of Infants; containing the general principles of their domestic treatment. 8vo. —J. Murray.

Tupper's (James Perchard) Essay on the Probability of Sensation in Vegetables; with additional Observations on Instinct, Sensation, Irritability, &c. 8vo.—White and Cochrane.

Morbid Anatomy of the Human Gullet, Stomach, and Intestines; illustrated with Twenty-one Engravings. By Dr. Alexander Monro, jun. Royal 8vo.—Longman and Co.

A Treatise on some Practical Points relating to the Diseases of the Eye. By the late John Cunningham Saunders; demonstrator of anatomy at St. Thomas's, Hospital, founder and surgeon of the London Infirmary for curing diseases of the eye.—To which is added, a short Account of the Author's Life, and his Method of Curing the Congenital Cateract. By his friend and colleague, J. R. Farr, M.D. Royal 8vo.

---

### *NOTICES TO CORRESPONDENTS.*

*We have been obliged, from want of space, to omit Communications from Messrs. I. R. Scott; Henry Smith; Andrew Blake; An Old Correspondent; An Essex Practitioner; T. E. B.; S. M.; &c. &c. Also an Account of Mr. Roberton's Treatise on Diseases of the Generative System.*

*We request our numerous Correspondents, in future, will address their Favors to the Publisher, Sir R. PHILLIPS, No. 47, Ludgate Hill; or to J. ADLARD, No. 23, Bartholomew Close.*

